# Supply chain and marketing of sea grapes, *Caulerpa racemosa* (Forsskål) J. Agardh (Chlorophyta: Caulerpaceae) in Fiji, Samoa and Tonga

**DOI:** 10.1007/s10811-014-0254-1

**Published:** 2014-02-16

**Authors:** C. Morris, S. Bala, G. R. South, J. Lako, M. Lober, T. Simos

**Affiliations:** 1 Institute of Marine Resources, School of Marine Studies, University of the South Pacific, Suva, Fiji; 2 School of Marine Studies, Faculty of Science, Technology and Environment, University of the South Pacific, Alafua, Samoa; 3 Faculty of Science, Technology and Environment, University of the South Pacific, Suva, Fiji; 4 Private Consultant, Apia, Samoa; 5 School of Agriculture, Food and Wine, University of Adelaide, Adelaide, Australia

**Keywords:** Harvesting, Marketing, Supply chain, Fiji, Samoa, Tonga

## Abstract

This report describes for the first time the supply chain of *Caulerpa racemosa* in three Pacific Island countries. The harvesting and marketing of *C. racemosa* are important subsistence activities for villagers in Fiji and Samoa, less so in Tonga. At least 150 harvesters are involved in Fiji, some 100 in Samoa and only a handful in Tonga. The annual combined crop is of some 123 t valued at around US$266,492. In Fiji, it is projected that supply does not meet local demand and there is a potential export market that is currently operating at a pilot project level. In Samoa, the supply is considered adequate for the current market. In Tonga, harvesting is carried out by a few families and supplies a niche market in that country. The possibilities of field cultivation of *Caulerpa* have been explored but, at present, with only limited success in Samoa. The supply chain is simple in all three countries, and only in Fiji are middlemen involved in the distribution process. The limitations for marketing include the fact that only a few sites supply most of the crop in all the three countries, that all sites need to be conserved through sustainable harvesting methods, the short shelf life of the crop and a lack of information on the carrying capacity of harvest sites. *Caulerpa* remains a crop that fulfils a niche market but has the potential to be scaled up for additional livelihood development in the future.

**Electronic supplementary material:**

The online version of this article (doi:10.1007/s10811-014-0254-1) contains supplementary material, which is available to authorized users.

## Introduction

The edible seaweed *Caulerpa racemosa* (Forsskål) J. Agardh (Class Bryopsidiophceae, Order Bryopsidales, Family Caulerpaceae) is widely consumed in the Pacific Islands and has been the subject of several reports on its harvesting and consumption in Fiji (South 1993a, 1993b, 1993c; South and Pickering 2006). Taxonomic studies on the genus *Caulerpa* were undertaken for Fiji and Samoa by South and N’Yeurt (1993) and South and Skelton (2003). The potential for the post-harvest treatment and export of *C. racemosa* was examined by Chamberlain (1997) and Chamberlain and Pickering (1996). The general market and food potential of *C. racemosa* were included in the reports of Novaczek (2001) and Pickering and Mario (1999). The supply chain of sea grapes in Fiji is described by Morris and Bala (2012). Previous reports have identified that the industry is based on harvesting mostly at the subsistence level, where the income derived supplements fishing, farming and other activities. There is no information available on the sustainability of the crop or the contribution that it makes to household incomes at the village level.

*C. racemosa* is one of 86 valid species worldwide (Guiry and Guiry 2011; www.AlgaeBase.org). In the tropical Pacific, there are more than 30 species, and many of them have a pantropical distribution. Fiji has a rich *Caulerpa* biodiversity, with 32 species known, one of which is endemic (South and Skelton 2003), but only 9 species have been reported from Samoa (Skelton and South 2007; Skelton and South 2014). The number of species found in Tonga is not yet recorded.

Although *Caulerpa* is widely consumed in the Pacific Islands and is the basis of a subsistence fishery, information on its supply chain, marketing and value is very scarce.

In this paper, we report on the supply chain in Fiji, Samoa and Tonga.

## Materials and methods

Supply chain and marketing surveys were carried out using semi-structured interviews based on questionnaires developed by fisheries personnel in Fiji and Samoa. The questionnaires were either translated into the local languages or were carried out by surveyors speaking in the local language (Annex 1). A total of 30 people were interviewed in the three target island countries.

The fisheries staff in Fiji and Tonga do not discriminate between *Caulerpa* and other inshore fishery products in their annual statistic reports. In Samoa, however, reliable data are collected and regularly maintained on a national Access database. The Samoa fishery survey methods include

**Fig. 1:**
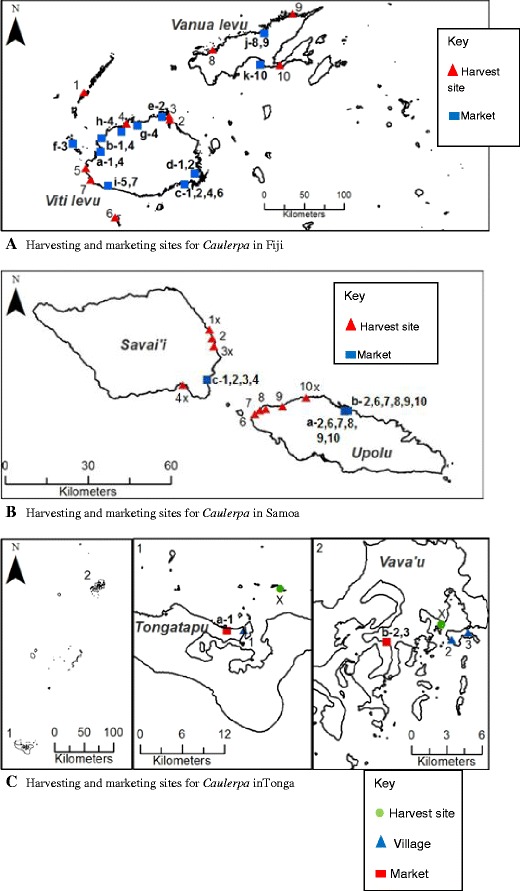
Supply chain maps for **a** Fiji, **b** Samoa and **c** Tonga, with the *numbers* representing the harvesting sites/villages (with exception to Tonga, where the harvesting sites are represented by the letter *X* and villages represented using *numbers*) and *alphabets* representing the marketing sites. Marketing sites are *hyphenated* with *numbers* indicating the source of seagrapes at respective outlets. Fig. 1a shows the Fiji harvesting sites/villages: *1* Gunu, *2* Namuimada, *3* Navolau, *4* Vatutavui, *5* Lomowai, *6* Vatulele, *7* Vusama, *8* Sasake, *9* Lakeba, *10* Dromoniku; and marketing sites: *a* Lautoka, *b* Nadi, *c* Suva, *d* Nausori, *e* Rakiraki, *f* Mana Island, *g* Tavua, *h* Ba, *i* Sigatoka, *j* Labasa, *k* Savusavu. Fig. 1b shows Samoa harvesting sites/villages: *1* Asaga, *2* Lano, *3* Faga, *4* Vailoa, *6* Satumalufilufi, *7* Mulifanua, *8* Lalovi, *9* Satapuala, *10* Leauva’a; and marketing sites: *a* Fugalei, *b* Apia fish market, *c* Salelologa, *x* roadside sales. Fig 1c shows Tonga harvesting villages: *1* Patangata, *2* Holeva, *3* Kaloa; and marketing sites: *a* Nuku’alofa, *b* Vava’u
- Vendors are asked which village they are from, how much seaweed they are selling on the day, and the price. The total production is estimated from a weighted average raised by a factor calculated regularly.

- Production data recorded from vendors at the Fugalei (Upolu) and Salelologa (Savaii) markets and the roadside, from surveys conducted three times a week. Roadside surveys are carried out on Upolu Island only. The bulk of harvested production each year is naturally sold at the Fugalei produce market in the capital followed by Salelologa market and least along the roadside (Fig 1).

Harvesting and supply chain data gathered from selected harvesting sites in Fiji, Samoa and Tonga were analysed using probability and trend analysis.

## Results

Detailed background information is provided in South et al.(2011); we are not aware of any previous studies on the supply chain and marketing of *C. racemosa* in the Pacific. We are also not aware of any work on the distribution, abundance and resource-carrying capacity of harvesting sites for *C. racemosa* anywhere in the Pacific. The impact of harvesting to determine long-term sustainability and capacity for further exploitation has not been investigated.

### Marketing, quantities and value

#### Fiji

The harvesting and marketing information for *Caulerpa* in Fiji is presented in Fig. 1A and Table 1. Approximately 75 % of the crop is from the Yasawa Islands. *Caulerpa* is sold in a number of markets, with the main municipal market located in Suva. In Fiji, *Caulerpa* is sold by portion (heaps), at prices ranging from FJ$2.00–4.00^1^ per heap, the weight of which ranges from 250–300 g. The plants are generally offered to customers on plastic plates, usually accompanied by a small plastic bag or cup of fermented coconut and fresh chilli. Whilst all municipal markets lack refrigeration facilities, Suva vendors make considerable effort to maintain freshness by splashing sea water on the bagged stock until ready for display.

**Table 1:** Annual production and revenue from *Caulerpa* harvesting and sales in Fiji in 2012

Site	Total Production per annum (t)	Total Revenue (FJD)
Yasawa	75.6	129,600
Sigatoka	2.7	24,300
Rakiraki	4.8	24,390
Tavua	9.0	27,000
Labasa	14.6	41,040
Savusavu	3.2	9,000
Total	110	FJD 255,330 (US$ 141,632)

The peak marketing days of *Caulerpa* in Fiji are Fridays and Saturdays to prepare for consumption on Sunday. Fresh, harvested stock arrives in the main markets (Suva and Lautoka) by Thursday afternoon. On Viti Levu, some harvesters do their own retailing but generally, most stock is sold direct to wholesalers and market vendors in Lautoka, Nadi, Sigatoka and Suva. In contrast, most harvesters located on Vanua Levu, Fiji's second largest island, retail their own stock directly with little wholesaling.

#### Samoa

A summary of harvesting and marketing sites, and production and value of *Caulerpa* in Samoa is shown in Fig. 1B and Table 2. Plants are also sold in heaps (known as *ofu limu* in Samoan) of up to 350 g in weight, wrapped either in foil or in breadfruit and coconut leaves. The selling price ranged from SAT$8 to 10 per heap (Lober 2011).

**Table 2:** Summary of production and value of *Caulerpa* harvested in Samoa in 2010

Site	Total Production per annum (t)	Total Revenue (SAT)
Leauvaa	3	44,133
Satapuala	0.76	11,913
Mulifanua	2.5	77,698
Satuimalufilufi	0.64	9,615
Lalovi	0.93	14,264
Total	7.83	157,623 (USD 69,196)

Source: Samoa Fisheries Division

Vendors in Samoa may sell between 40 and 80 heaps per week. Transport to market is usually by a combination of bus and ferry boat where orders are required to be sourced from the island of Upolu. Orders from caterers and hotels occur occasionally, and hoteliers and restaurateurs pick up their own orders from harvesters in Savai’i. At Lano, harvesters supply orders for Upolu caterers and hoteliers usually up to 20 *ofu limu* at one time. These are taken by bus to Salelologa wharf by the harvester or a relative, then by a combination of ferry boat to Upolu, bus to Fugalei market and finally taxi to its final destination. Costs associated with this supply method amount to SAT$48.00, inclusive of return modes of transport (fares by bus, ferry and taxi).

According to fisheries database records, *Caulerpa* was harvested from a total of 47 villages on Upolu and Savaii islands from 2005 to 2010 which showed a stable average trend. Although a stable value was maintained over the last 4 years, the first 2 years showed a lower value. The lowest value of *Caulerpa* in 2006 (SAT$91,337) equated to smaller harvests, and this shortfall in supply led to an increased average price of SAT$15 kg^−1^ in later years.

#### Tonga

A summary of the harvesting and marketing sites, and quantities and value of the crop is presented in Fig. 3 and Table 3. *Caulerpa* is sold in only two municipal markets in Tonga, Nuku’alofa and Vava’u Islands. There are only two families from the village of Patangata supplying the Nukualofa market on a daily basis (except Sunday). The two families have only one harvesting site which is the reef flat between Onevai and Onevau Island. The harvesters sell their own produce and there are no middleman involved.

**Table 3:** Summary of production and value of *Caulerpa* in Nuku’alofaTonga in 2012

Site	Total production per annum (t)	Revenue per annum (TOP)	Average value per kg (TOP)
Nuku’alofa (Tongatapu)	2.5	41, 433	17
Vava’u	0.9	5,760	6
Total	3.4	47,193 (USD 26,735)	14

There are two villages involved in *Caulerpa* harvesting and sales in Vava’u: the Holeva Village with two families involved and the Koloa village with only one family involved. Unlike Nukualofa, sales are only done in the weekend, whereby the family from Koloa sells on Fridays and Saturdays all year around, and the families from Holeva sells on Saturdays but only for 6 months (during peak season) per year.

### The market and supply chains

**Fig. 2:**
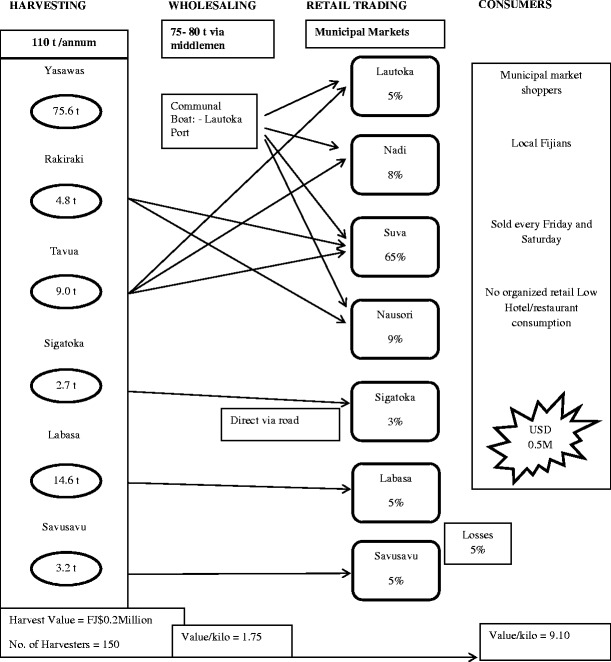
Industrial supply chain map for *Caulerpa*—Fiji

**Fig. 3:**
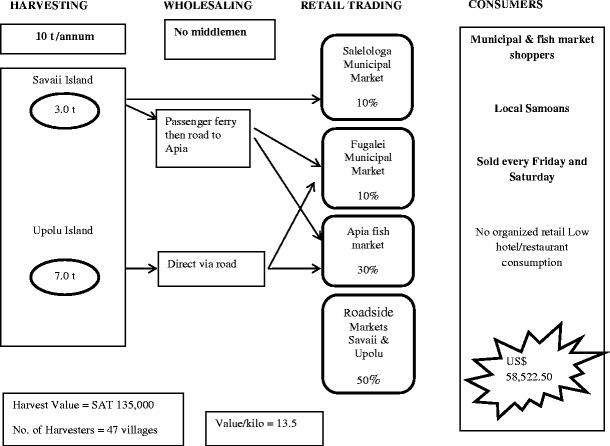
Industrial supply chain map for *Caulerpa*—Samoa

**Fig. 4:**
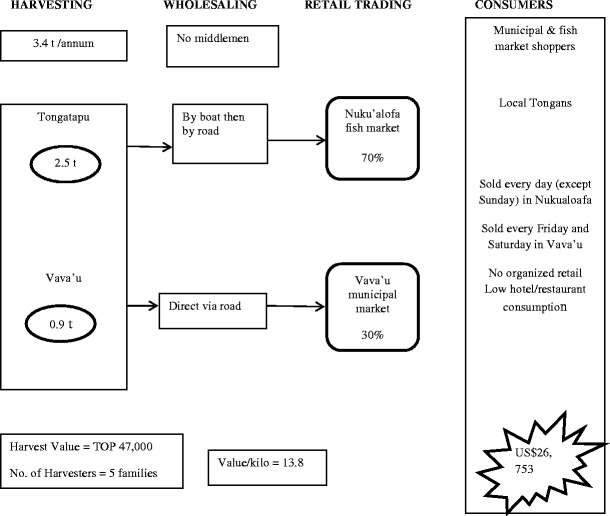
Industrial supply chain map for *Caulerpa*—Tonga

The supply chains in Fiji, Samoa and Tonga are shown in Figs. 2, 3 and 4 and Tables 1–3, respectively. From surveys conducted at ten sites in six areas of Fiji, a total of some 150 harvesters (part-time and full-time) were recorded. Production ranged from 5 to 2,100 kg week^−1^, with an average of 323 kg week^−1^. The main production areas were the Yasawa Islands, followed by Labasa, Tavua and Rakiraki.

*Caulerpa* harvesting and sales provide a part-time subsistence occupation for many villagers in Fiji and Samoa, with a potential income of more than FJ$200 per week for harvesters. The industry provides only a small proportion (<1.0 %) of the overall national income from the inshore fishery, and *Caulerpa* sales are overshadowed by sales of other seaweeds in Fiji (South 1993b).

According to Bala and Finau (2012), the annual production in Tonga was 3.4 t (Nuku’alofa 2.5 t, Vava’u 0.9 t) with a market value of TOP47,193 (Table 3).

## Discussion

The combined annual *Caulerpa* crop for Fiji, Samoa and Tonga amounts to some 123 t, valued at US$266,492. There are some important aspects of the industry that need to be examined. The carrying capacity of the main harvesting beds has not been studied, and the population density and biomass at these sites are not yet known. The careful conservation of harvesting sites (as in the Yasawa Islands in Fiji) over long periods of time by local villagers may be a good measure allowing predictable harvesting. Sustainable harvesting (i.e. not removing the runners) is another important practice carried out by most harvesters in Fiji and Samoa, but not in Tonga. The introduction of improved post-harvest treatment by the use of proper wound-healing technology would prolong the life of the crop from harvesters to consumers.

The fact that harvesting of *Caulerpa* is limited to a few main sites where the resource grows readily and is easily and safely accessible to harvesters in coastal communities may mean that the industry is potentially vulnerable to loss of product. This loss may be due to the combined impacts of unsustainable harvesting and natural phenomena such as storm surges and cyclones, resulting in the sites becoming unproductive. A preliminary result from an ongoing Fiji biomass survey has shown that this has been the case with Rakiraki. Conservation of sites is to a certain extent in the hands of the harvesters themselves, but they are not formally protected. Harvesting is part of a traditional activity which for most includes inshore gleaning for shells, sea cucumber and fishing for the daily family meal.

Compared with other commodities, the supply chains are simple, and only in Fiji are middlemen routinely used. The crop goes directly to market with a 3-day shelf life due to limitations of being able to provide wound healing as a post-harvest process. Shelf life could be substantially increased if harvesters were to use an appropriate wound-healing methodology such as holding in aerated seawater for up to 48 h following harvesting. Roadside sales do not routinely occur in Fiji, but they are common in Samoa on both of the main islands. The lack of sustainable harvesting at some sites is a potential threat to the long-term survival of the beds. The market supply chains show that the loss of crop occurs during handling between harvesters and consumers. This is partially a result of inadequate quality control. The port and the market serve as the consolidation point for middlemen who then carry out the distribution to other local markets and roadside stalls and in some case to some resorts.

Sustainability of the *Caulerpa* industry in the three island countries will depend on collection of more data with regards to carry capacity of important harvesting sites, identification of new potential sites for both wild and farm harvests and through raising awareness and training both harvesters and fisheries officers on management issues. In Samoa, some pilot trials to cultivate the local *C. racemosa* have been carried out on Upolu Island in collaboration with Samoa fisheries (Dr. N Paul, personal communication 2011). This method uses 1-m^2^ shallow trays with double layers of netting on the bottom: the *Caulerpa* runners are placed between the two layers, allowing the shoots to grow through when the trays are anchored in shallow water.

Given the short shelf life and the many critical control points determined during preliminary surveys along the chain (especially since markets are up to 100 km away), it would be worthwhile to conduct a proper Hazard Analysis and Critical Control point (HACCP) analysis of the cold chain in order to determine the best methods of intervention. This would be the focus of future research.

## Electronic supplementary material

Below is the link to the electronic supplementary material.

ESM 1 (DOCX 16 kb)

## Footnotes

1 Approximate exchange rates: FJD1.00 = US$0.559; SAT1.00 = US$0.439;TOP 1.00 = US$0.55.
