# Supplementary material for: Supply chain and marketing of sea grapes, Caulerpa racemosa (Forsskål) J. Agardh (Chlorophyta: Caulerpaceae) in Fiji, Samoa and Tonga
Source: J Appl Phycol. 2014 Feb 16;26(2):783–9. doi: 10.1007/s10811-014-0254-1 (PMC3988519; doi:10.1007/s10811-014-0254-1)
Supplement: Supplementary file 1 — (DOCX 16 kb) [file 10811_2014_254_MOESM1_ESM.docx]

**Annex 1. Questions for seagrape interviews:**

**­­­­­­­­­­­­_______________________ Village, __________________Country**

1. How are seagrapes harvested and how many people from the village is involved? How is it stored after harvest? Is there any cost involved in harvesting: - boat etc.?
2. Have you noticed any difference in seagrape coverage over the years? How many weeks does it take for the same area to recover for the next harvest; crop rotation?
3. Is there any history behind seagrape collection. How was it started in the village? Has seagrapes been there since time?
4. Any medicinal benefits of seagrapes which you know of?
5. How many bags can one woman collect per day? Specify bag size and time consumed to fill the bag (CPUE).
6. How is the sales money distributed?
7. In total, how many women are involved in seagrape harvest and what percentage are these to the number of house hold in the whole village?
8. How may bags collected per week per village?. Estimate total harvest for the month and year?
9. Is there seasonality of seagrape harvesting (collect more/less in different months)?
10. Is there different types of seagrapes found?
11. If Yes, then describe these.
12. Which type of seagrape is better and why:- what qualities do they have, any taste difference, customer preference (demand), get different names (local names).
13. Is there different price for different types?
14. Which type stays fresh for longer?
15. Can harvesters negotiate a better price for fresh seagrapes?
16. How is seagrapes sold? If middlemen are involved: - get details of middle man:- where are they from, how many are there, how much they pay; how they transport; any idea where they retail; etc
17. Price for 1 bag (size of bag; weight of bag)
18. If harvesters do not selling seagrapes in market, what options are there for them to sell. Would they prefer to sell themselves and get more money, or would they rather sell to middleman? WHY?
19. Do the villages sell other products in the markets and to the middleman?
20. If harvesters sell themselves, then how are seagrapes transported to the market, what are the cost involved, how is it stored during transportation?
21. What is the harvesters view on the demand of seagrapes? Do they think, they can sell more, if proper transport is available, or they think that what they are selling is sufficient?
22. If sold on early week days:- would people buy?
23. Who all are the consumers; Chinese, Indo-Fijians, Fijians: - who buys more?
24. Is seagrapes from other areas sold in the respective markets. If yes, then where from?
25. Is there any information of the Hotels usage of seagrapes, if YES then who sells to them? If possible get contact details.
26. How is seagrapes prepared, any new recipes?
27. Is there any scope of expanding the seagrape industry?
